# Correspondence

**Published:** 1878-10

**Authors:** George T. Moffatt

**Affiliations:** Boston


### CORRESPONDENCE.

BOSTON, July 16, 1878.

TO THE EDITOR OF THE DENTAL REGISTER:—The time of annual meeting of the American Academy of Dental Science has been changed from the last Monday in September to the last Wednesday in October, believing this to be a more favorable time for securing a large attendance. Members and others will please take notice of this change.

Please insert this in your next number and oblige, yours truly,
GEORGE T. MOFFATT,
Cor. Sec. Am. Acad. Dental Science.

